# Immunomodulatory Expression of Cathelicidins Peptides in Pulp Inflammation and Regeneration: An Update

**DOI:** 10.3390/cimb43010010

**Published:** 2021-05-13

**Authors:** Nireeksha, Sudhir Rama Varma, Marah Damdoum, Mohammed Amjed Alsaegh, Mithra N. Hegde, Suchetha N. Kumari, Srinivasan Ramamurthy, Jayaraj Narayanan, Eisha Imran, Juzer Shabbir, Zohaib Khurshid

**Affiliations:** 1 Department of Conservative Dentistry and Endodontics, A. B. Shetty Memorial Institute of Dental Sciences, NITTE (Deemed to Be) University, Mangalore, Karnataka 575018, India; drnireeksha@nitte.edu.in (N.); drhegdedentist@gmail.com (M.N.H.); 2 Department of Clinical Sciences, College of Dentistry, Ajman University, Ajman 4599, United Arab Emirates; marahdamdoum@gmail.com; 3 Center of Medical and Bio-Allied Health Sciences Research, Ajman University, Ajman 4599, United Arab Emirates; 4 Department of Oral and Craniofacial Health Sciences, College of Dental Medicine, University of Sharjah, Sharjah 27272, United Arab Emirates; mohammedalsaegh@yahoo.com; 5 Department of Biochemistry, K. S. Hegde Medical Academy, NITTE (Deemed to Be) University, Mangalore, Karnataka 575018, India; suchetha.shetty@rediffmail.com; 6 College of Pharmacy and Health Sciences, University of Science and Technology of Fujairah, Fujairah 2202, United Arab Emirates; s.ramamurthy@ustf.ac.ae; 7 Department of Basic Sciences, College of Dentistry, University of Science and Technology of Fujairah, Fujairah 2202, United Arab Emirates; j.narayanan@ustf.ac.ae; 8 Department of Dental Biomaterials, HITEC Dental College, Taxila 47080, Pakistan; eishaimran@ymail.com; 9 Department of Operative Dentistry, Liaquat College of Medicine and Dentistry, Karachi 75290, Pakistan; dr.juzer.shabbir@gmail.com; 10 Department of Prosthodontics and Implantology, College of Dentistry, King Faisal University, Al-Ahsa 31982, Saudi Arabia; zsultan@kfu.edu.sa

**Keywords:** cathelicidins, dental pulp, inflammation, dental caries, innate immunity

## Abstract

The role of inflammatory mediators in dental pulp is unique. The local environment of pulp responds to any changes in the physiology that are highly fundamental, like odontoblast cell differentiation and other secretory activity. The aim of this review is to assess the role of cathelicidins based on their capacity to heal wounds, their immunomodulatory potential, and their ability to stimulate cytokine production and stimulate immune-inflammatory response in pulp and periapex. Accessible electronic databases were searched to find studies reporting the role of cathelicidins in pulpal inflammation and regeneration published between September 2010 and September 2020. The search was performed using the following databases: Medline, Scopus, Web of Science, SciELO and PubMed. The electronic search was performed using the combination of keywords “cathelicidins” and “dental pulp inflammation”. On the basis of previous studies, it can be inferred that LL-37 plays an important role in odontoblastic cell differentiation and stimulation of antimicrobial peptides. Furthermore, based on these outcomes, it can be concluded that LL-37 plays an important role in reparative dentin formation and provides signaling for defense by activating the innate immune system.

## 1. Introduction

The dental pulp undergoes various stages of repair and regeneration throughout its functioning as a result of microbial activity and other insults. The repair and regeneration process mainly occurs due to molecular signaling pathways, inflammatory cascades, and immunomodulatory effects [1]. The local environment of the pulp responds to any changes in physiology that are highly fundamental, such as odontoblast cell differentiation and other secretory activity [2]. Presenting host cell damage and maintaining a sterile pulpal environment is one of the aims of pulpal health. The external factors that affect the pulpal environment include dental caries, physical, mechanical, and chemical irritation, and microbial infection [3].

The histology of reversibly injured dental pulp shows an absence of bacteria and localized coagulation necrosis, while in inflamed pulp, it exhibits the infiltration of inflammatory cells such as neutrophils, suggesting chemotaxis [2,4]. These inflammatory cells release lysosomal enzymes that cause tissue destruction, leading to permanent, irreversible damage, or necrosis [5], requiring endodontic treatment with/without placement of intracanal medicaments to achieve complete disinfection and the prevention of postendodontic pain [6,7]. Various signaling events in the pulp stimulate various mediators, which play vital roles in the progression of inflammation or result in abrupt regression of the inflammatory process [8]. T-lymphocytes are essential residents of dental pulp. Initial accumulation of CD^3+^ T-lymphocytes, plasma cells, and neutrophils around pulpal dendritic cells are usually seen [9]. The interaction between the dendritic cells and T-lymphocytes results in the mobilization and activation of different cells, thereby causing immuno-pathological events. The increased influx of plasma cells, the production of immunoglobulin, and the perivascular presence of dendritic cells leads to immune response [9]. This increased patrolling by memory T-lymphocytes and macrophage-derived cytokines IL-1, IL-6, and TNF-α, as well as upregulation of the adherence molecules intercellular adhesion molecule 1 (ICAM-1), vascular cell adhesion molecule (VCAM-1), and endothelium-leukocyte adhesion molecule (ELAM-1) on the surfaces of endothelial cells, explains the inflammatory process in the pulp [9].

**Figure 1:**
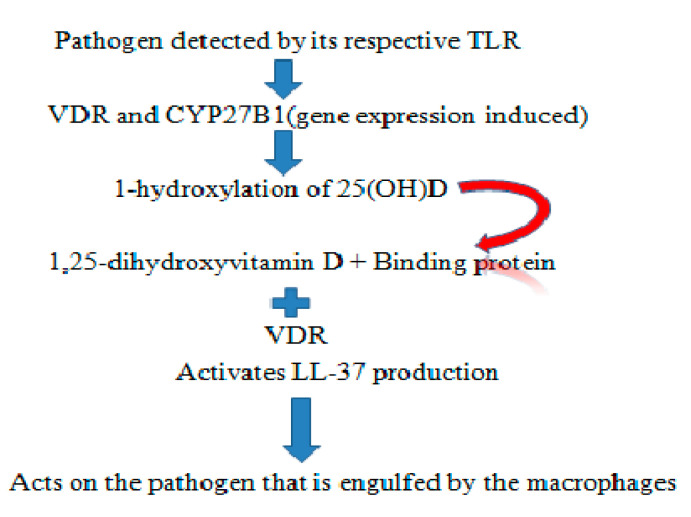
Representation of the TLR2/1–Vitamin D cathelicidins (LL-37) pathway.

Cathelicidins are antimicrobial peptides naturally occurring in saliva [9,10], gingival crevicular fluid (GCF) [11], and blood. They possess antimicrobial and antiviral capabilities, which can be useful for formulating drugs. The oral cavity is an exceptional environment for microorganisms to quickly gain access. The widespread distribution of cathelicidins results in action against microorganisms [12]. Antimicrobial peptides can include anionic peptides, which are small and rich in glutamic acids and aspartic acid, although LL-37 from humans are linear cationic α-helical peptides [13,14]. Anionic and cationic peptides containing one or more disulphide bonds, like protegrin from pigs, tachypleins from horse grabs, and α-β-defensins from humans, are similar to antimicrobial peptides like lactoferrin [15]. The LL-37 antimicrobial peptide is regulated by the *CAMP* gene. A known inducer of *CAMP* gene expression is 1,25 dihydroxyvitamin-D through Vitamin-D receptor binding to the Vitamin-D responsive element at 500 bp upstream of the *CAMP* gene promoter [16]. The first experiment to prove LL-37 peptide expression upregulation, to fight against bacterial infection was carried out using a cystic fibrosis xenograft model, where a planned overexpression of LL-37 using an adenovirus vector was performed [17]. A promising candidate for inducing LL-37 is 1,25-dihydroxyvitamin D3 through Toll-like receptor via the TLR2/1- Vitamin D Cathelicidins (LL-37) pathway (see Figure 1).

In endodontics, these cathelicidins are addressed based on their capacity to heal wounds, their immunomodulatory potential, and their ability to stimulate cytokine production and recruit immune-inflammatory response in pulp and periapex by regulating reparative dentin formation through the stimulation of odontoblasts [18]. These peptides are highly potent therapeutic agents against viral, bacterial, and fungal pathogens. These synthetic peptides, which possess unique properties, are also referred to as peptidomimetics, and evolved from contemporary research endeavors [19]. A synthetic peptide like VS2, VSL2, has also proven to be bactericidal against endodontic pathogens and decreases bacterial load to a depth of 400 µm [20].

Antimicrobial peptide LL-37 and its potential role in innate immunity, dental caries pathogenesis, periodontal health, and other oral inflammatory conditions have been evaluated, and a number of factors with relation to clinical evidence are available. It is essential to focus on their oral health potential, and specifically on dental pulp inflammation [21,22]. Therefore, this review aims to assess the role of cathelicidin (LL-37) peptides in dental pulp, and their role in immuno-defense mechanisms and reparative dentin formation.

## 2. Brief Literature Search

This narrative review highlights a brief non-systematic methodology to include the selected articles for discussion.

## 3. Methods and Materials

Accessible electronic databases (Medline, Scopus, Web of Science, SciELO, and PubMed) were used to find studies that reported the role of cathelicidin in pulpal inflammation and regeneration published between September 2010 and September 2020. The following MeSH terms were used for brief searching of the data bases: “Cathelicidins”, “Dental pulp”, and “inflammation”. Articles in English language, in vitro cell line studies, ex vivo studies, and clinical trials explaining the role of LL-37 peptide in pulpal inflammation and dental caries were included in this review.

## 4. Results

Five articles deemed eligible are discussed in Table 1.

## 5. Discussion

### 5.1. Structure and Function of LL-37

Understanding the structure and function of these peptides is essential and well established. They are released biochemically in two different prokaryotes; they are ribosomally synthesized and, in particular, within mammalian leukocytes; the granules hold large amounts of antimicrobial peptides precursor molecules [26]. Cathelicidins are a structurally and functionally distinct protein class as part of innate immunity in mammals.

These peptides were first isolated from bovine neutrophils called Bac5, further cloning cDNA to reach the gene [27]. The hallmark of these peptide families is the presence of highly preserved cathelicidin domain. It was termed ‘alarmins,’ referring to its capacity to modulate inflammation, limiting damage to host cells, improving wound healing, angiogenesis, and eliminating abnormal cells [28]. LL37/hcap18 is the only known cathelicidins referred to as ‘Pleotrophic,’ ‘Multifaceted,’ ‘Multifunctional,’ and ‘factotum’ [29].

**Figure 2:**
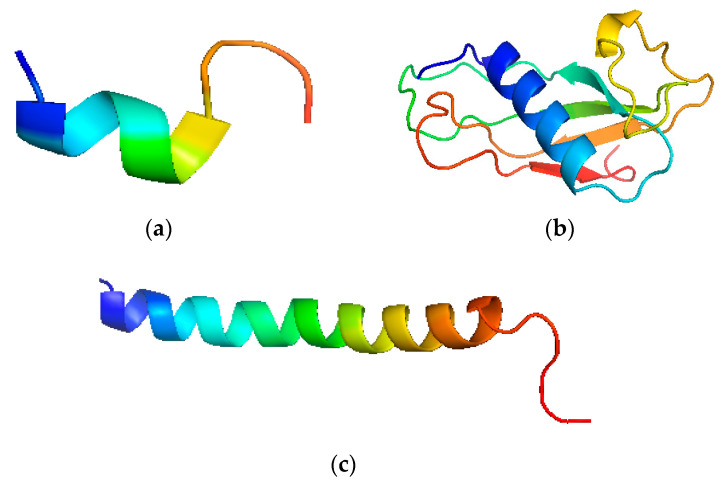
Pictorial depiction of LL-37 structure. (**a**) N-terminal fragment of LL-37, (**b**) Cathelin like the domain of human cathelicidins, (**c**) C-terminal fragment of LL-37.

The structure has a highly conserved N-terminal domain with 100 amino acid residues. It has N-terminal and an antimicrobial domain. It is an 18 kDa protein, the primary product after translation is called Pre-protein, which is a prelude to cathelicidin holo-protein [30] (Figure 2). The single cleaved protein further targets the cathelicidins to the storage granules or the cell exterior. This form is called inactive or storage form. It further cleaves into the cathelin domain and participates in host activities [31] (Figure 2).

LL-37 belongs to α-helical A.M’P.’s. They are present in different cells, tissues, and body fluids at varying concentrations, mainly saliva [32], wound fluids [33], gingiva [34], leukocytes [35], and squamous epithelial cells [36]; its presence is also enhanced within intracellular fluids in case of minor inflammation and infection. The action of these antimicrobial peptides is present against *Streptococcus Mutans* group A, B, C, *Staphylococcus Aureus* [37], *E.faecalis* [38], *Lactobacillus acidophilus* [39], *pseudomonas aeruginosa*, *Aggregatibacter Actinomycetumcomitans, Candida Albicans*, spirochetes, and yeasts [29].

Cathelicidins show a high-affinity binding property to lipopolysaccharides. It has the virulence to neutralize L.P.S. [30]. LL-37 contributes to angiogenesis and wound healing along with promoting epithelial cell proliferation and transactivation of epidermal growth factors [31]. Biofilm inhibition is also achieved in many ways by preventing attachment of bacterial cells, improvising quorum sensing system, and down-regulation of gene promoting biofilm formation [32]. Some of the significant activities of cathelicidins on host cells are forming transient pores on the cellular membranes, causing bacterial cell dissociation, leakage of components, transactivation of receptors, interacting with binding sites, neutralization of microbial lipopolysaccharides released from damaged host cells, and initiating pro-inflammatory response [33]. Hence, cathelicidins are clearly a part of innate immunity, whereby they decrease microbial adhesion, and are also a potential part of dental caries pathogenesis.

### 5.2. Antimicrobial Peptide Cathelicidins and Caries

The primary etiological cause of dental caries is predominantly the presence of *streptococcus mutans* [35]. The components of unstimulated and stimulated saliva that flow or bathes the tooth surface inhibits the adhesion, colonization and binding of these bacteria [36]. These peptides are bactericidal and overcome bacterial resistance mechanisms [37]. Various A.M’P.’s detected in saliva, namely H.N.P. 1-3, LL-37, defensins, and their expression dictates the growth, proliferation, and survival of different microbial species [38]. Antimicrobial peptides in saliva provide immediate action against destructive microbial species, thereby providing optimal protection against caries progression, prevents overgrowth of microorganisms, and simultaneously maintaining a stable ecology system [39].

The source for salivary antimicrobial peptide LL-37 is salivary gland and ductal cells [40]. The relationship of dental caries and LL-37 peptide has been studied in middle school children, which showed significantly higher levels of LL-37 in the no caries group. The study concluded that the expression of these peptides is associated with caries prevalence [23]. In the estimation of LL-37 in unstimulated whole saliva among children of the age group 2-18 years old, the analysis of LL-37 was found to be low in children with high caries activity compared to children with low and moderate caries activity, suggesting that LL-37 is a vital protector molecule of immunity in the oral cavity [21,22]. A study conducted to evaluate the effect of synthetic peptides β-defensins hBD2,hBD3, and LL-37(Cap18) on oral bacteria, namely *A.A.comitans, P.gingivalis, F.nucleatum, S.Mutans, S.Sanguis, S.Mitis,* and *L.casei* showed LL-37 and β-defensins have versatile antibacterial activity against oral bacteria [26]. Antimicrobial peptides play a potential role in pulp protection by odontoblastic stimulation, innate immunity, and reparative dentin formation.

### 5.3. Dental Pulp, Innate Immunity and Cathelicidins

The pathogen recognition receptors (P.R.R.) functions as a part of innate immunity functioning in the dental pulp. The family of P.R.R. includes C-type lectin receptors, Toll-like receptors, nucleotide-binding oligomerization domain-like receptors, and AIM2 like receptors that cause immunomodulation [41]. Studies also assess the role of specialized immune cells like cytokines, IL-8, which up-regulates and recruits neutrophils to the inflammation site [42]. This suggests a balance between the inflammatory and repair process in the pulp. IL-6, TNF -α, distribution of human telomerase-derived peptide, and downregulation of L.P.S. induced inflammatory cascades also contribute to this process [1]. The immunocompetent cells located in the periphery of the pulp, namely the odontoblasts, encounter a vast microbial array and have an immense capacity of orchestrating inflammatory response. Odontoblasts have a significant role to play in innate immunity and environmental sensing [4]. These molecular mediators are locally produced and initiate various cellular events that facilitate upregulation and downregulation of specific peptides, creating a specialized environment in pulpal low compliance space [4].

Odontoblasts play a significant role due to reasons like (a) the odontoblastic process that extend into the dentinal tubules making them the first cell to recognize and encounter microorganisms and their bacterial products by penetrating through enamel and dentin [43]; (b) they present antimicrobial peptide [44]; (c) these odontoblast form pseudo epithelial layers that are partially impermeable barrier [45]; (d) they are closely associated with dendritic cells, lymphocytes and thereby respond to any injury to dentin through caries/mechanical/chemical injury [46]; and (e) in response to lipopolysaccharides, they produce IL-8 and stimulate neutrophils chemotaxis to the area of inflammation, all of which suggest a substantial role in immune defense [47]. The Toll-like receptors (T.L.R.) are the primary class of microbial recognition receptors. Activation of T.L.R. regulates the production of antimicrobial peptides, chemokines, cytokines, leukocytes regulation, T cell function, basically providing a bridge between innate and adaptive immunity [48]. The odontoblasts differentially recognize and respond to Gram-negative and Gram-positive bacteria through T.L.R. 2 and T.L.R. 4 utilization and expression [49]. This is further justified by a study that showed that T.L.R. 2 and T.L.R. 4 are prominently distributed at the odontoblast cell body interface and the dentin layer. These validations suggest the efficiency of odontoblasts to attract neutrophils, antimicrobial peptides, and pro-inflammatory cytokines [50]. Pulpal diseases are caused by various bacteria that reside in a low compliance environment within the pulp, creating inflammation and infiltration of mediators like neutrophils, immune cells, and molecules expressed in the cascade of inflammation that serves as biomarkers [51]. The presence of other regulatory inflammatory molecules may also affect the inflammatory response. The identification of biomarkers typically plays an essential part in understanding stages of pulpal inflammation [52].

Pulp tissues were studied by RT-PCR, multiplex assay, microarray, Western blot, radioimmunoassay, immuno-histochemistry, Enzyme-linked-immunosorbent-assay (ELISA), zymography, and flow cytometry [26]. A total of 64 biological markers showed a statistically significant difference between inflamed and healthy pulp [4]. This showed an active presence of antimicrobial peptides in pulp tissue by the RT-PCR technique method, targeting mRNA, which functions as part of the innate and adaptive immune system [18]. To detect the expression of LL-37 in a dental pulp diagnosed with symptomatic irreversible pulpitis and apical periodontitis, it was compared with healthy pulp [53]. In the tooth diagnosed for symptomatic irreversible pulpitis with apical periodontitis, pulp chamber deroofing was done, and pulpal blood samples were collected with paper points and further analyzed for LL-37 levels with ELISA [53]. The mean value of LL-37 in normal pulp was 0.2 ± 0.6 ng/mL and in symptomatic irreversible pulpitis showed 1.5 ± 1.2 ng/mL, which clearly showed high levels of LL-37 in infected dental pulp [53].

Further, it was hypothesized that the expression of LL-37 is due to neutrophil infiltration and its expression in inflamed pulp tissue [18]. Thus, LL-37 is currently discussed in endodontic literature based on its ability to promote human pulp cell migration, resulting in the regeneration of the pulpal dentin complex and dentinal bridge formation. Hence, the potential role of LL-37 can probably be utilized for healing inflamed pulp [54].

To assess the role of odontoblasts in innate immunity by the production of LL-37, an odontoblast-like cell line was obtained from mouse pulp tissue, which structurally and functionally displayed odontoblasts. The effect of bacterial endotoxins L.P.S. from Gram-negative bacteria and L.T.A. from Gram-positive bacteria on the MDPC-23 cell line was studied by assessing IL-6, MMP-8, and LL-37(CRAMP) expression. The odontoblasts possess immune-like cell properties and modulate the innate immunity system. The expression of LL-37 by odontoblasts was associated with reparative dentin formation and innate immunity system via these mechanisms [55].

The antimicrobial peptide LL-37 is present in different cells, tissues, and body fluids, in mature neutrophils and specific granules as pro-proteins bound to plasma lipoproteins which are an important reservoir for LL-37. The up-regulation of these peptides suggests their role in the immune system to battle against inflammatory conditions. Various bacterial products stimulate the production of antimicrobial peptides expression; the expressed peptides perform a diverse role in hampering/slowing down the inflammatory process/disease process [56,57]. Koczulla and co-workers conducted a study to emphasize the role of LL-37 in the neovascularization of the rabbit model [57]. Any wound healing scenario, vascularization, or formation of new blood vessels were essential factors observed in the study. The mediators of inflammation stimulate the formation of new capillaries and enrichment of already persisting vessels [58]. Angiogenic potential of LL-37 peptide was evaluated in a study where human umbilical cord endothelial cells were coated on dishes at different concentrations of LL-37, which was used as a stimulant. The follow-up was done for 18hr. ELISA estimated the VEGF levels in the containers. The study concluded that peptide LL-37 induced angiogenesis due to its direct activity on endothelial cells [57].

The effective induction of angiogenesis may be a significant factor in healing ulcers, wounds, and any inflammatory condition [33]. In vivo application of LL-37 showed vessel growth in physiological and pathological angiogenesis models. A G-protein coupled receptor FPRL1 mediates this cellular response on biding to peptide LL-37 [59]. The pathways that elicit endothelial activation are PLC-γ/PKC/NF-κB, the Erk-1 and -2 MAPK, and the PI3K/Akt factors. LL-37 always attracts neutrophils and monocytes in vivo to the target site [60]. These cells contain various amounts of angiogenic mediators [56]. The pathway is initiated by densely present cells in areas of inflammation or wound sites due to increased expression of LL-37. Thus, the presence of the peptide analogues activates vascular endothelial growth factor [61].

The above mechanism justifies that LL-37 can be an effective candidate for stimulating angiogenesis in pulp [62,63]. LL-37 peptides are considered unstable and easily dissociated by bacteria and host proteases in vivo. The LL-37 with heparin as a complex exerts a bactericidal effect on various oral microbes. Once in contact with the microorganism, LL-37 dissociates and performs relevant actions accordingly [58]. In dental pulp, the heparin LL-37 complex permeates dental pulpal tissue when applied as a pulp capping agent. This complex causes the destruction of the bacterial cell membrane as proposed by carpet, barrel stave, and toroidal models [59]. The regulatory effect on angiogenesis, vascular endothelial growth factor expression in the hDPC’s, immune responses, cellular migration, and bone regeneration can be enhanced by the high dose of LL-37 in cytoplasm without any membrane lytic action that may promote pulp tissue repair [53].

Morbus Kotsmann is a syndrome exhibiting congenital neutropenia. Neutrophils store various potential microbicidal effectors, namely LL-37, α-defensins, and H.N.P. 1-3 [55]. These individuals are treated with granulocytes colony-stimulating factor (G-CSF), which improves the neutrophil count and quality of patients’ lives. Furthermore, even in instances when the neutrophils were in an average amount, infection persisted in these patients, postulating that neutrophils were deficient in antimicrobial peptide, LL-37. The individuals who suffered from Kotsmann Syndrome have poor periodontal health and chronic periodontal disease. The study strongly suggested that the LL-37 has an in vivo role in bacterial infections [60].

**Figure 3:**
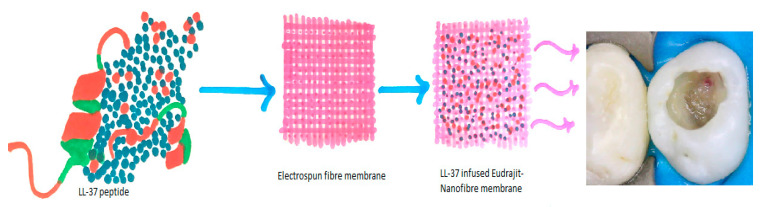
Pictorial representation of LL-37 in electrospun fiber membrane as a potential pulp capping agent.

The LL-37 peptides stimulate the migration of UDMSC’s (Undifferentiated mesenchymal cells) in pulpal inflammation, bone repair, and reparative dentin formation. Efforts were made to elaborate on the effect of LL-37 on cell viability, which suggests a favorable proliferation of cell lines [61]. Ten µg/mL of LL-37 stimulates dentin sialoprotein and dentin phosphoprotein in dentin mineralization [62]. Therefore, the role of antimicrobial peptides in odontoblast differentiation further corroborates its role as a significant dentinogenesis marker in favorable conditions, which is further validated in this study [62]. However, the study suggests conducting further research in understanding the complete process of odontoblastic differentiation and dentin deposition, which can shed light on pulpal differentiation [18]. Cathelicidins serve as potent mineralization agents. The peptides on the tooth surface prevent demineralization of enamel by acids produced through colonized bacteria [53]. The role of antimicrobial peptides in stimulating odontoblast’s for dentin deposition and preventing bacterial biofilm formation makes these peptides a new tool in the array of synthetic peptides to promote dentin formation and prevent the occurrence of dental caries and periodontal destruction. This potential of LL-37 peptide can be utilized as a pulp capping agent, where this synthetic peptide acts as a precursor for odontoblastic cell differentiation and dentin bridge formation [53] (Figure 3).

The studies selected showed few drawbacks, but offer a potential area for scope in justifying LL-37 role in decreasing bacterial count, stimulating odontoblastic differentiation, and as a therapeutic agent for pulpal protection [59,61,62]. The role of LL-37 synthetic peptide in inhibition of various resident bacteria in the oral cavity showed scope for understanding, if there is selective inhibition that takes place when treated with LL-37 peptide [57,63]. A study evaluating levels of LL-37 in middle-aged children showed varying levels of beta-defensins (Cathelicidins), whereas alpha-defensins could be used as caries risk assessment factor. The study further provides scope for understanding if the expression of LL-37 can be altered by external stimulation [21,22]. The migration of stem cells of the apical papilla with various concentrations of synthetic peptides showed its efficiency to stimulate, but in vivo studies could help in better understanding the effect of these peptides on apical lesions and proliferation of pulpal cells. The studies selected evaluated LL-37 expression in the inflamed and normal pulp, but were done with smaller sample size. To prevent bias, studies can be done using a larger sample size to evaluate levels of LL-37 expression, and the rate of inflammation required for expression of LL-37 also needs to be evaluated (Table 1).

This review’s limitation is the presence of few clinical studies on the potential application of LL-37 on dental pulp inflammation. More in vivo clinical studies would enhance the evidence and could further validate the effect of LL-37 in pulpal health and inflammation.

## 6. Conclusions

Cathelicidins (LL-37) have a significant role in reparative dentin formation and its potential role in the pulp’s innate immune defense system. Potential therapeutic agents can be prepared using commercially available LL-37 peptides as a pulp capping agent. The presence of limited biomarkers in modulating the actions of dentinal cells by signaling pathways has been a challenge to determine the role of peptides conducive to pulpal regeneration. Further studies evaluating the role of these peptides in pulpal inflammatory conditions may open a new array of diagnostic ideas in preserving pulpal health, assessing hematogenous flow, and limiting conventional treatment in minimal inflammatory diseases of the dental pulp. The role of these peptides can offer a promising start in evaluating cells related to pulpal regeneration and proliferation.

## Figures and Tables

**Table 1:** Characteristics of included studies.

Author	Experimental Area	Methodology	Outcome	Conclusion	Remarks
Sarmiento et al., 2016 [18]	To identify LL-37 levels in normal and inflamed pulp	10 diagnosed symptomatic irreversible pulpitis with apical periodontitis and 10 Healthy pulp were chosen. Estimation of LL-37 was done by ELISA method.	Mean pulpal concentration of LL-37 in symptomatic irreversible pulp is 1.5 ± 1.2 ng/mL and in normal pulp is 0.2 ± 0.6 ng/mL.	Inflammation of dental pulp induces expression of LL-37 ad therefore LL-37 plays a major role in innate immunity.	Small sample size selection might be a bias to standardize the levels of LL-37 in inflamed and normal pulp. The degree of inflammation of pulp required to stimulate LL-37 expression is another factor that needs to be assessed.
Tao et al., 2005 [23]	To identify possible relationship between Antimicrobial peptide levels in saliva and caries experience in middle school children	Saliva of 144 children under the age group of 11–15 yrs were analyzed for LL-37 and hBD3, HNP1-3.	The mean of LL-37 showed extensive variation between individuals. The level of (α-defensins) HNP1-3 were inversely correlated with caries.	Children with caries have significantly lower levels of α-defensins and low salivary levels of H.N.P 1-3 was inversely correlated to caries	The study showed alpha-defensins can be potential markers for caries risk. Also mentioned that synthetic peptides can be developed to enhance peptide expression and can be therapeutic agent.
Ouhara et al., 2005 [22]	Anti microbial peptide hBD1, hBD2, hBD3 and LL-37 were evaluated for their antimicrobial activity against oral bacteria	Synthetic peptides were treated with various oral bacteria *Aggregatibacter actinomycetemcomitans, Porphyromonas gingivalis, Prevotella intermedia, Fusobacterium nucleatum, Streptococcus mutans, Streptococcus sobrinus, Streptococcus salivarius, Streptococcus sanguis, Streptococcus mitis and Lactobacillus casei*	Antibacterial action of hBD1 was lower than other peptides *F. nucleatum* was highly susceptible to hBD 3 and LL-37. *S. mutans* was highly susceptible to hBD3	β-defensins and LL-37 have potential antibacterial action against oral bacteria	The net charge on the bacterial surface is another factor that can be considered, also selective inhibition by these peptides can be a contradictory factor that can be evaluated through invivo studies.
Cheng et al., 2020 [24]	Evaluate the effect of LL-37 on proliferation, migration and differentiation of Stem cells from apical papilla (SCAP)	SCAPs were isolated from third molars of age group 16–20 Y, cultured, characterized. The cell viability was analyzed with cell-commet assay kit. Dentin Sialophosphoprotein, dentin matrix protein 1 were assessed by quantitative polymerase chain reaction and western blot.	2.5 µg/mL of LL-37 regulated odonto/osteogenic markers. LL-37 promoted alkaline phosphatase activity	LL-37 at 2.5 µg/mL promoted the migration and odonto/osteo differential SCAPs by activating the AKt/Wnt/β-catenin signalling	In vivo assay could have been a better option, to understand therapeutic action of LL-37 on apical lesions.
Khung et al., 2015 [25]	To examine in-vitro effects of LL-37 on expression of vascular endothelial growth factors in human pulp cells	Pulp cells at passage 6 were treated with 10 µg/mL^−1^ synthesized LL-37 and inhibition assay was performed with MAPk or NF-kB inhibits VEGF, mRNA, VEGF protein andphophorylated ERK were determined byRT PCk, ELISA and western blot	LL-37 significantly increased both mRNA and protein levels VEGF in pulp cell.	LL-37 activated the ERK pathway to boost VEGF secretion from human pulp cells. These for can be a potent pulp capping agent.	This article clearly suggests the role of LL-37 in pulpal cell migration and thus a potent pulp capping agent.

## Data Availability

Not applicable.
